# Clinical Presentations of Celiac Disease: Experience of a Single Italian Center

**DOI:** 10.3390/nu17010129

**Published:** 2024-12-31

**Authors:** Chiara Maria Trovato, Francesca Ferretti, Anna Pia Delli Bovi, Giovanna Elefante, Monica Ancinelli, Giulia Bolasco, Teresa Capriati, Sabrina Cardile, Daniela Knafelz, Fiammetta Bracci, Arianna Alterio, Monica Malamisura, Salvatore Grosso, Paola De Angelis, Antonella Diamanti

**Affiliations:** 1 Nutritional Rehabilitation Unit, Bambino Gesù Children Hospital IRCCS, 00165 Rome, Italy; francesca.ferretti@opbg.net (F.F.); monica.ancinelli@opbg.net (M.A.); giulia.bolasco@opbg.net (G.B.); teresa.capriati@opbg.net (T.C.); sabrina.cardile@opbg.net (S.C.); daniela.knafelz@opbg.net (D.K.); fiammetta.bracci@opbg.net (F.B.); arianna.alterio@opbg.net (A.A.); antonella.diamanti@opbg.net (A.D.); 2 Clinical Pediatrics, Department of Molecular Medicine and Development, University of Siena, 53100 Siena, Italy; delliboviannapia@gmail.com (A.P.D.B.); elefantegiovanna76@gmail.com (G.E.); salvatore.grosso@unisi.it (S.G.); 3 Gastroenterology and Nutrition Unit, Bambino Gesù Children Hospital IRCCS, 00165 Rome, Italy; monica.malamisura@opbg.net (M.M.); paola.deangelis@opbg.net (P.D.A.)

**Keywords:** celiac disease, clinical presentation, Italy, gastrointestinal symptoms, diagnosis, gluten, autoimmune disease, prevalence

## Abstract

Background/Objectives: In Italy, the prevalence of celiac disease (CeD) among children exceeds 1.5% and has steadily increased with a linear trend over the past 25 years. The clinical presentation is heterogenous and a change in onset symptoms has been described in recent years. The aim of the study is to describe the pattern of clinical presentation of CeD during the last 12 years in a single Italian center. Methods: We retrospectively enrolled all children diagnosed with CeD at Bambino Gesù Children Hospital, Rome between 1 March 2011 and 22 June 2023. To investigate the changes in pattern of clinical presentation, we divided the patient population into three groups of approximately 4 years each (respectively: 49, 48 and 48 months). Patients who previously received a CeD diagnosis in other centers were excluded. Results: Overall, 4478 patients were diagnosed with CeD at our center. 1082 were excluded, leaving 3396 available for analysis. We divided the study cohort into three groups: group 1 (*n* = 909), group 2 (*n* = 1103), and group 3 (*n* = 1384). Diagnoses of CeD increased by 17.5%. The trend of the non-classic form shows a significative increase (*p* = 0.000064), showing a high prevalence of bloating and abdominal pain and a significant reduction in celiac crisis (*p* < 0.0001). Conclusions: Annual diagnoses of CeD increased during the study period, and the clinical presentation has changed in recent years, showing an increase in the non-classic form and a reduction in more severe forms of celiac crisis.

## 1. Introduction

Celiac disease (CeD) is an autoimmune enteropathy triggered by gluten intake, arising in genetically predisposed individuals. It is currently estimated to have a worldwide prevalence of 0.7–1.4% based, respectively, on serology and biopsy, with a female predominance and an almost double incidence in the pediatric population [1]. In Italy, the prevalence of celiac disease among children exceeds 1.5% and has presented a steady increase with a linear trend over the past 25 years [2]. A recent multi-center cross-sectional study conducted in Italy [3] reported a 1.65% overall prevalence of celiac disease (CeD) in the country, with the highest prevalence recorded in the southern regions of the island at 1.93%. Despite this, celiac disease remains a largely underdiagnosed condition with a 70% rate of misdiagnosed cases [4]. One of the prominent causes of such alarming rates is the heterogeneous clinical manifestation; in fact, in addition to typical gastrointestinal symptoms, many cases begin with atypical manifestations due to extra-intestinal signs, or even without any symptoms. The classification currently used is based on the Oslo criteria [5]. Based on these criteria, “typical forms” are usually present in younger children as failure to thrive, chronic diarrhea, abdominal distention, muscle wasting and hypotonia, poor appetite, and unhappy behavior; “atypical forms” are characterized by unusual intestinal complaints (vomiting) or by extra-intestinal manifestations (e.g., pubertal delay, iron deficiency, aphthosis, dental enamel defects, and abnormalities in liver function). In conclusion, asymptomatic patients are usually screened for associated conditions such as diabetes mellitus type 1, autoimmune thyroiditis, epilepsy, Down Syndrome or a family history of CeD. Recent evidence [6,7,8,9] has shown a change in clinical presentation with a decreasing presence of malabsorption symptoms in the onset clinical picture.

The aim of this study is to describe the pattern of clinical presentation of CeD in Italian children during the last 12 years, diagnosed in a single tertiary clinical center, highlighting any difference from the past.

## 2. Materials and Methods

We retrospectively enrolled all children diagnosed with CeD at tertiary care academic hospital Bambino Gesù Children Hospital, Rome between 1 March 2011 and 22 June 2023. All patients were diagnosed according to current ESPGHAN guidelines [10,11,12]. To investigate the changes in pattern of clinical presentation, we divided the patient population into three groups of approximately 4 years each (respectively: 49, 48 and 48 months). Patients who previously received a CeD diagnosis in other centers were excluded. For all patients, demographic and anthropometric characteristics, symptoms, laboratory tests and antibodies values, and familiarity for CeD or other autoimmune diseases were collected. Symptoms were collected according to Oslo Classification.

We excluded patients who had previously received a CeD diagnosis in other centers and those with missing data.

### Statistical Analysis

Statistical analysis was performed using the statistical software R (version 4.2.2, R Core Team, Vienna). Furthermore, Pearson’s Chi-square was used to measure whether prevalence of the different symptoms changed according to time of diagnosis. Significance was set at *p* < 0.05.

## 3. Results

**Figure 1:**
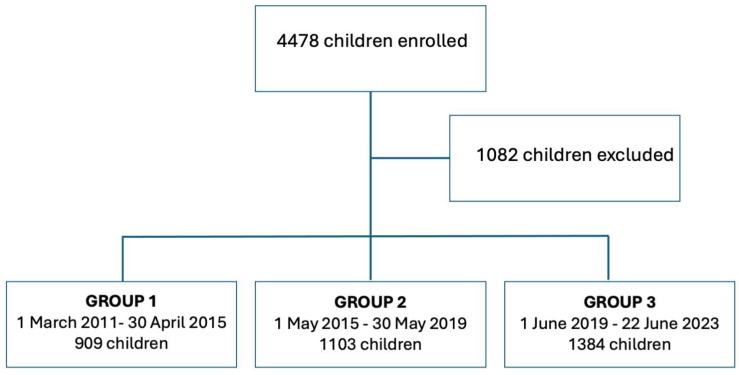
Flowchart of study population.

The study initially included 4478 patients who visited Bambino Gesù Pediatric Hospital for their first consultation regarding celiac disease. Of these, 1070 were excluded due to a prior diagnosis at another center. Additionally, 2 patients were excluded because of missing data, and 10 others were excluded because their diagnosis fell outside the study period (Figure 1).

Ultimately, 3396 patients (female = 2131, median age = 6.9 years old, age range = 8 months−18.8 years) were enrolled in the study cohort. The study population was divided in three groups, according to the diagnosis date:

- Group 1 (*n* = 909): patients who received a diagnosis from 1 March 2011 to 30 April 2015;
- Group 2 (*n* = 1103): patients who received a diagnosis from 1 May 2015 to 30 May 2019;
- Group 3 (*n* = 1384): patients who received a diagnosis from 1 June 2019 to 22 June 2023.

Out of 3396 patients, 2314 (68.1%) patients received a diagnosis with biopsy-sparing protocol, according to ESPGHAN 1990, ESPGHAN 2012 or ESPGHAN 2020 criteria [10,11,12]. Of the 1082 biopsied children, Marsh–Oberhuber grade 3 lesions were present in 989 patients, Marsh–Oberhuber grade 2 lesions in 42 patients, and Marsh–Oberhuber grade 1 lesions in 51 patients, respectively.

The rate of diagnosis of celiac disease increased by 52.2% from the period 2011–2015 to the period 2019–2023.

**Figure 2:**
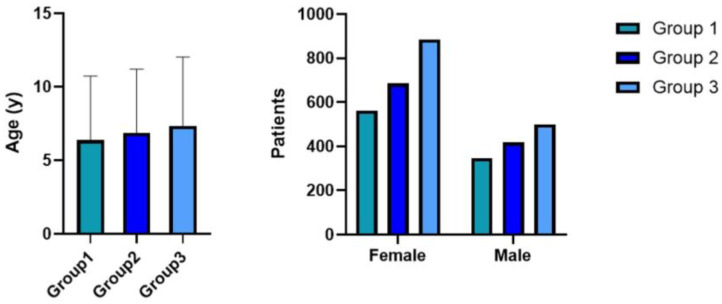
Distribution of age and sex at diagnosis in the three groups.

Group 1 comprised 562 females (61.8%) and 347 males (38.2%), with a median age of 6.4 years. Group 2 included 685 females (62.1%) and 418 males (37.9%), with a median age of 6.9 years. Group 3 consisted of 885 females (63.9%) and 499 males (36.1%), with a median age of 7.3 years (Figure 2). A significant increase in age at diagnosis among the three groups was observed (F (2, 3391) = 13.18, *p* < 0.0001).

Conversely, no significant differences were found in the distribution of males and females among the three groups (χ²(2) = 1374, *p* = 0.508).

Clinical features of the study cohort were classified according to Oslo criteria. Overall, 2305 patients (67.8%) debuted with a classic form, with signs and symptoms of malabsorption, while 1006 patients (29.6%) debuted with a non-classic form. 85 patients out of 3396 (2.5%) showed an asymptomatic presentation.

The percentage of the classic form in the three groups was 69% (*n* = 629), 70% (*n* = 770) and 65% (*n* = 906), respectively; the percentage of the non-classic form was 27% (*n* = 249) in group 1, 29% (*n* = 320) in group 2 and 31.6% (*n* = 437) in group 3. The chi-square statistic was 17.36 with a *p*-value of 0.0016.

**Figure 3:**
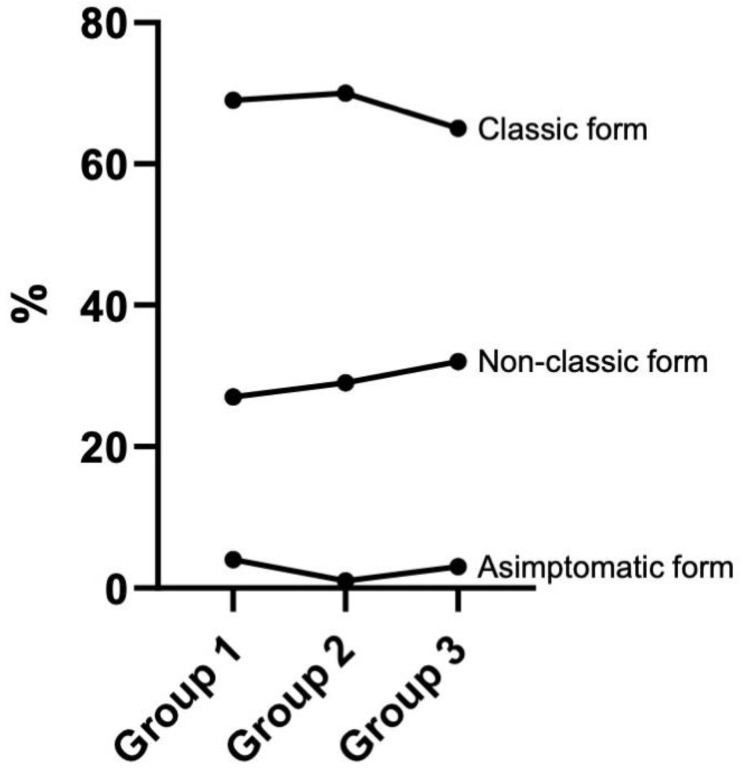
Classic, non-classic and asymptomatic forms variation during the time. Group 1, *n*= 909, diagnosed between 1 March 2011 and 30 April 2015; group 2, *n*= 1103, diagnosed between 1 May 2015 and 30 May 2019; group 3, *n*= 1384, diagnosed between 1 June 2019 and 22 June 2023.

In order to observe the trend of non-classical form across the three-time periods, the Cochran Armitage trend test was also applied. The results (statistic-z = 3.99; *p*-value = 0.000064) confirm a significant difference (Figure 3).

The distribution of single symptoms is reported in Table 1. Bloating and abdominal pain showed a notable variation, indicating that the prevalence of these symptoms differ significantly among the three groups. Similarly, weight loss, failure to thrive, iron deficiency anemia, asymptomatic presentation, and celiac crisis were also significant for *p* < 0.05. Conversely, several symptoms did not exhibit statistically significant differences among the groups, presenting a substantial stability over time. Group 1 and group 3 were also compared individually, yielding consistent results in most cases. However, for symptoms such as constipation and oral stomatitis, a significant increase was observed from Group 1 and group 3. A reduction in celiac crisis was also observed. In group 1 a total of 33 children (3.6%) presented a celiac crisis at the onset of disease, while in group 3 the percentage decreased to 0.8% (11 children with celiac crisis out of 1384 diagnoses of celiac disease), overtaken by group 2 with a percentage of 1.2%. The statistical analysis using the chi-square test revealed a χ^2^ value of approximately 28.15 (*p*-value < 0.00001).

Table 2 summarizes diagnoses of CeD in other associated conditions. Regarding other autoimmune diseases, 2% of new celiac disease diagnoses were made in patients with type 1 diabetes and 1% in patients with rheumatoid arthritis. The incidence rate has remained stable over the analyzed years, as well as the associated syndromes.

## 4. Discussion

Diagnosis of celiac disease has continued to increase in recent years. Recent data show that the prevalence of CeD in Italy, estimated between 2017 and 2020, was 1.65% [3]. Data in the literature show an increase in the number of annual diagnoses [7]; we likewise observed a percentage growth of celiac diagnosis of 52% from 2011–2015 to 2019–2023. The reasons behind this partly lie in improved screening methods and partly in the increase of true pathology due to changes in environmental factors and dietary habits [13]. It can also be argued that awareness of CeD is also increasing, thanks to media attention directed to this topic [14]. Additionally, the average age at diagnosis has shifted from under 2 years to 6–9 years in many developed countries [15]. Our findings corroborate this trend, with an average diagnosis age of 7 years in our cohort from 2019 to 2023.

In recent decades, a change in clinical presentation is occurring. New symptoms previously unrelated to CeD are now recognized as part of a broader spectrum of manifestations. As an example, neurological disorders such as migraines and ataxia are now considered among “non-classical” symptoms in children [16], and there is a growing interest in other manifestations in adult CeD, such as infertility and sexual dysfunction [17,18,19]. A retrospective study on the Dutch population, conducted from 2007 to 2016, showed a shift in the clinical spectrum of presenting symptoms in pediatric CeD towards an atypical presentation [6]. A North American retrospective study, including patients from 1994 to 2014, showed a light prevalence of the non-classic form of CeD (43%) compared to the classic form (34%) or asymptomatic form diagnosed by screening (23%) [7]. A recent Spanish study [20] examined the clinical presentation between 2011 and 2017 and found that, while the classical form remained the most common, its prevalence significantly declined over the study period. These findings align with our results, which indicate an increase in non-classical presentations at the expense of classical forms, although the latter remains the most frequent.

Considering each symptom individually, gastrointestinal issues are the most prevalent. However, their prevalence is shifting over time. A study conducted in western New York showed that abdominal pain and constipation were the most common symptoms in newly diagnosed celiac patients and that these symptoms occur more frequently in children older than CeD children with classic presentation [21]. In a multicentric study conducted in the US, abdominal pain was reported in 57% of the study cohort [22]. In Greece, patients presenting with malabsorption signs were less than 45%; among the most frequent onset symptoms were constipation, recurrent abdominal pain, and vomiting [8]. An Italian study [9] recently analyzed CeD clinical presentation in children for 30 years, reporting a reduction in diarrhea and an increase in constipation and bloating. Our results confirm the prevalence of gastrointestinal symptoms (i.e., intestinal meteorism, 77.6%, diarrhea, 34%, abdominal pain, 54.5%). In agreement with Pedretti et al. [9], in this study the prevalence of intestinal meteorism and constipation increased, while diarrhea did not show a significant difference during the study period analyzed. Other symptoms demonstrate a decrease over time, such as iron deficiency anemia, weight loss, and failure to thrive. Moreover, a reduction in the incidence of celiac crisis was observed, underlining a greater attention by both clinicians and families to the appearance of warning symptoms of celiac disease. It is important to remember that celiac crisis is a rare presentation of CeD, although it can occur at any age, including in elderly patients [23]. Moreover, regarding clinical presentation, it is important to not forget the “strange” and rare form of presentation as a dermatologic sign (e.g., acrodermatitis [24] and pemphigus vulgaris [25]).

Recent data show an increase in the incidence of autoimmune diseases; our results show a stable percentage of co-occurrence of type 1 diabetes and CeD. A strict GFD is beneficial for the long-term management of these autoimmune conditions, as shown by a decrease in the serum concentration of different autoantibodies [26]. In light of the upcoming national screening program of T1DM and CeD, this figure is likely to change significantly [27,28]. On top of T1DM, several other autoimmune diseases should be assessed and included in CeD evaluation and screening, ranging from autoimmune thyroid disease to autoimmune hepatitis [29,30].

Our study has several strengths, including the large sample size and the extended duration of data collection and observation. At the same time, the main limitations of this study are its retrospective design and the involvement of only one center.

## 5. Conclusions

The increasing diversity in the clinical presentation of CeD highlights the critical importance of recognizing and diagnosing this condition. The established shift towards atypical symptoms needs increased awareness among healthcare providers to ensure timely identification and management. With new screening campaigns on the horizon, we can anticipate a further rise in celiac disease diagnoses in the coming years. This underscores the need for continuous education and vigilance, not only among clinicians but also within the general public, to recognize the wide spectrum of symptoms associated with CeD. Addressing this growing awareness is crucial for improving patient outcomes and enhancing the quality of life for those affected by this condition.

## Figures and Tables

**Table 1:** Clinical presentation of celiac disease. Gr1 = group 1; Gr2 = group 2; Gr3 = group 3; χ^2^ (G1-G2-G3) = chi square derived from the comparison among the three groups; χ^2^ (G1-G3) = chi square derived from the comparison between group 1 and group 3.

Symptoms	*n*	Group 1 (*n* = 909)	Group 2 (*n* = 1103)	Group 3 (*n* = 1384)	χ^2^ (G1-G2-G3)	χ^2^ (G1-G3)
Diarrhea	1159	332 (36.5%)	386 (35%)	441 (31.8%)	χ^2^ = 5.84 *p* = 0.053	χ^2^ = 5.33 *p* = 0.021
Bloating	2636	629 (69.1%)	909 (82.4%)	1098 (79.3%)	χ^2^ = 54.05 *p* < 0.00001	χ^2^ = 30.32 *p* < 0.00001
Abdominal pain	1853	526 (57.8%)	650 (58.9%)	677 (48.9%)	χ^2^ = 30.28 *p* < 0.00001	χ^2^ = 46.20 *p* < 0.00001
Vomiting	931	236 (25.9%)	321 (29.1%)	356 (35.7%)	χ^2^ = 4.10 *p* = 0.128	χ^2^ = 0.016 *p* = 0.8978
Constipation	1017	248 (27.2%)	328 (29.7%)	441 (31.8%)	χ^2^ = 5.52 *p* = 0.063	χ^2^ = 7.55 *p* = 0.006
Weight loss	703	219 (24%)	207 (18.7%)	277 (20%)	χ^2^ = 9.27 *p* = 0.009	χ^2^ = 5.38 *p* = 0.0204
Failure to thrive	1650	464 (51%)	576 (52.2%)	610 (44%)	χ^2^ = 19.31 *p* = 0.00006	χ^2^ = 10.69 *p* = 0.0011
Change in mood	852	250 (27,5%)	270 (24.4%)	332 (24%)	χ^2^ = 3.92 *p* = 0.140	χ^2^ = 3.58 *p* = 0.0586
Iron deficiency anemia	1166	341 (37.5%)	418 (37.8%)	407 (29.4%)	χ^2^ = 25.18 *p* < 0.00001	χ^2^ = 16.39 *p* = 0.00001
Hypertransaminasemia	153	37 (4%)	55 (4.9%)	61 (4.4%)	χ^2^ = 1.023 *p* = 0.599	χ^2^ = 0.15 *p* = 0.6963
Recurrent aphthous stomatitis	212	69 (7.6%)	70 (6.3%)	73 (5.3%)	χ^2^ = 5.058 *p* = 0.079	χ^2^ = 5.064 *p* = 0.0244
Dental enamel defects	210	45 (4.9%)	102 (9.2%)	63 (4.5%)	χ^2^ = 26.58 *p* < 0.00001	χ^2^ = 0.194 *p* = 0.6596
Celiac crisis	58	33 (3.6%)	14 (1.2%)	11 (0.8%)	χ^2^ = 28.15 *p* < 0.00001	χ^2^ = 23.43 *p* < 0.00001
Asymptomatic	85	31 (3.4%)	13 (1.2%)	41 (2.9%)	χ^2^ = 12.19 *p* = 0.0022	χ^2^ = 0.362 *p* = 0.5475

**Table 2:** Associated conditions.

Associated Condition	*n*	Group 1 (*n* = 909)	Group 2 (*n*= 1103)	Group 3 (*n* = 1384)
Type 1 Diabetes	213	45 (4.9%)	102 (9.2%)	66 (4.7%)
Chronic autoimmune thyroiditis	86	45 (4.9%)	17 (1.5%)	24 (1.73%)
Rheumatoid arthritis	32	12 (1.32%)	5 (0.45%)	15 (1%)
Down Syndrome	40	7 (0.77%)	18 (1.63%)	15 (1%)
Turner Syndrome	5	1 (0.11%)	4 (0.36%)	0
Other Syndromes	7	1 (0.11%)	3 (0.27%)	3 (0.2%)

## Data Availability

Data for the present study are available from the corresponding author upon reasonable request.
